# Exploring Biomarkers in Breast Cancer: Hallmarks of Diagnosis, Treatment, and Follow-Up in Clinical Practice

**DOI:** 10.3390/medicina60010168

**Published:** 2024-01-17

**Authors:** Laura Lopez-Gonzalez, Alicia Sanchez Cendra, Cristina Sanchez Cendra, Eduardo David Roberts Cervantes, Javier Cassinello Espinosa, Tatiana Pekarek, Oscar Fraile-Martinez, Cielo García-Montero, Ana María Rodriguez-Slocker, Laura Jiménez-Álvarez, Luis G. Guijarro, Soledad Aguado-Henche, Jorge Monserrat, Melchor Alvarez-Mon, Leonel Pekarek, Miguel A. Ortega, Raul Diaz-Pedrero

**Affiliations:** 1 Department of Surgery, Medical and Social Sciences, Faculty of Medicine and Health Sciences, University of Alcalá, 28801 Alcala de Henares, Spain; laura.lgonzalez@uah.es (L.L.-G.); anarodriguezslocker@gmail.com (A.M.R.-S.); soledad.aguado@uah.es (S.A.-H.); raul.diazp@uah.es (R.D.-P.); 2 Ramón y Cajal Institute of Sanitary Research (IRYCIS), 28034 Madrid, Spain; oscarfra.7@hotmail.com (O.F.-M.); cielo.gmontero@gmail.com (C.G.-M.); luis.gonzalez@uah.es (L.G.G.); mademons@gmail.com (M.A.-M.); leonel.pekarek@gmail.com (L.P.); miguel.angel.ortega92@gmail.com (M.A.O.); 3 Oncology Service, Guadalajara University Hospital, 19002 Guadalajara, Spain; ali.96sc@gmail.com (A.S.C.); csc.orbis@gmail.com (C.S.C.); edy_roberts@hotmail.com (E.D.R.C.); jcassinelloespinosa@gmail.com (J.C.E.); 4 Department of Medicine and Medical Specialities, Faculty of Medicine and Health Sciences, University of Alcalá, 28801 Alcala de Henares, Spain; tatianapekarek@gmail.com (T.P.); laura.jimenezal@gmail.com (L.J.-Á.); 5 Department of General and Digestive Surgery, General and Digestive Surgery, Príncipe de Asturias Universitary Hospital, 28805 Alcala de Henares, Spain; 6 Unit of Biochemistry and Molecular Biology, Department of System Biology (CIBEREHD), University of Alcalá, 28801 Alcala de Henares, Spain; 7 Immune System Diseases-Rheumatology, Oncology Service an Internal Medicine (CIBEREHD), University Hospital Príncipe de Asturias, 28806 Alcala de Henares, Spain; 8 Cancer Registry and Pathology Department, Principe de Asturias University Hospital, 28806 Alcala de Henares, Spain

**Keywords:** breast cancer, biomarkers, serological, histological, translational medicine

## Abstract

Breast cancer is a prevalent malignancy in the present day, particularly affecting women as one of the most common forms of cancer. A significant portion of patients initially present with localized disease, for which curative treatments are pursued. Conversely, another substantial segment is diagnosed with metastatic disease, which has a worse prognosis. Recent years have witnessed a profound transformation in the prognosis for this latter group, primarily due to the discovery of various biomarkers and the emergence of targeted therapies. These biomarkers, encompassing serological, histological, and genetic indicators, have demonstrated their value across multiple aspects of breast cancer management. They play crucial roles in initial diagnosis, aiding in the detection of relapses during follow-up, guiding the application of targeted treatments, and offering valuable insights for prognostic stratification, especially for highly aggressive tumor types. Molecular markers have now become the keystone of metastatic breast cancer diagnosis, given the diverse array of chemotherapy options and treatment modalities available. These markers signify a transformative shift in the arsenal of therapeutic options against breast cancer. Their diagnostic precision enables the categorization of tumors with elevated risks of recurrence, increased aggressiveness, and heightened mortality. Furthermore, the existence of therapies tailored to target specific molecular anomalies triggers a cascade of changes in tumor behavior. Therefore, the primary objective of this article is to offer a comprehensive review of the clinical, diagnostic, prognostic, and therapeutic utility of the principal biomarkers currently in use, as well as of their clinical impact on metastatic breast cancer. In doing so, our goal is to contribute to a more profound comprehension of this complex disease and, ultimately, to enhance patient outcomes through more precise and effective treatment strategies.

## 1. Introduction

Breast cancer, recognized as the most prevalent cancer worldwide, is also the leading cause of cancer-related mortality among women in both developed and developing nations. As of 2022, this disease accounted for approximately 3 million new cases globally, which constituted roughly 13% of all cancer diagnoses. During the same period, it led to more than 600,000 deaths [1]. Specifically, breast cancer represents 25% of all neoplasms in women, with an overall incidence rate of 50 per 100,000. However, this incidence rate varies, as evidenced by Belgium’s notably higher rate of 113 cases per 100,000 women [2,3].

Over recent decades, there has been a significant 30–40% rise in the incidence of breast cancer. This increase can primarily be attributed to the widespread adoption of mammography screening programs, especially targeting women in higher-risk age groups. Consequently, these initiatives have enabled an earlier detection of the disease, undeniably improving patient prognosis [4].

Regarding risk factors, several have been identified as particularly significant. Age, for instance, emerges as a crucial factor, with women over 50 facing a greater risk. Furthermore, a family history of breast cancer, as well as a previous diagnosis in males, increases this risk. Genetic factors also play a role, with mutations in genes such as BRCA1, BRCA2, TP53, CDH1, PTEN, and STK11 significantly raising the risk [5,6]. Additionally, lifestyle factors such as obesity, long-term use of hormone replacement therapies (including birth control pills and HRT), and excessive alcohol consumption, particularly among women aged 55 and older, have been linked to an increased risk of developing metastatic breast cancer [7,8,9].

In terms of screening and early detection, the U.S. Preventive Services Task Force (USPSTF) plays a pivotal role. As per their 2021 guidelines, they recommend that women of average risk begin biennial mammography screening at age 50 and continue until age 74 [10]. The diagnostic process typically starts with a thorough physical examination and breast imaging tests like mammography or ultrasound to identify any suspicious lumps or areas of thickening. If a lump is detected, the next step usually involves a needle biopsy to confirm the presence of cancer cells. Additional diagnostic tools, such as MRI for high-risk patients and CT or PET scans, help to further understand the extent of the cancer and its potential spread [11,12].

Regarding the stage at diagnosis, about 64% of women are found to have localized cancer, 27% have regional involvement, and 6% are diagnosed at an advanced stage. Identifying the specific type of cancer is heavily reliant on histological and pathological markers [13,14]. Moreover, molecular classification, which involves evaluating hormone receptors and HER2/neu expression, is critical for determining both therapeutic approaches and prognostic outcomes [15,16].

The treatment of metastatic breast cancer is intricately tailored based on these molecular markers. For example, in HER2-negative metastatic tumors with positive hormone receptors, the first-line treatment involves hormone blockade combined with aromatase inhibitors or antiestrogens, often paired with a CDK 4/6 inhibitor like palbociclib. In contrast, for triple-negative tumors, assessing PDL1 status is essential to decide on the use of atezolizumab; otherwise, chemotherapy is considered, though it offers limited benefits [17]. The role of BRCA1 and BRCA2 expression in these patients cannot be overstated, as it greatly influences the choice of targeted therapy, such as poly ADP-ribose polymerase inhibitors. In the case of HER2-positive metastatic tumors, the first-line treatment typically includes trastuzumab-, pertuzumab-, and taxane-based chemotherapy [18]. It is important to note that the treatment landscape for metastatic breast cancer is continually evolving, with ongoing clinical trials showing the effectiveness of new therapies, like the combination of conjugated antibodies with chemotherapy [19].

Finally, during the course of metastatic disease, follow-up imaging tests and the monitoring of serological tumor markers are crucial for detecting any signs of recurrence. This allows for timely adjustments in systemic treatment based on peripheral blood samples [20]. The emergence of new biomarkers, including circulating tumor cells and genetic markers, is increasingly playing a vital role in the diagnosis and prognosis of breast cancer, further enhancing the management of and treatment outcomes for patients with advanced stages of the disease.

## 2. Luminal A

The designation “luminal” for certain breast cancer subtypes arises from their gene expression profiles, which bear a resemblance to the luminal epithelium of the breast. These cancers typically express luminal cytokeratins 8 and 18. Among these subtypes, the most prevalent and the one associated with the best prognosis is Luminal A breast cancer. This subtype accounts for approximately 40% of all breast cancer cases [21]. Luminal A breast cancer is characterized by high expression levels of genes associated with hormonal receptors and low expression of the HER2 gene group, coupled with a low proliferation gene signature.

### 2.1. Histological Biomarkers

Hormone receptors play a pivotal role as primary biomarkers in luminal breast cancer. There are two primary forms of the estrogen receptor (ER), namely ERα and ERβ. However, only ERα holds a validated clinical role, being expressed in 70–75% of breast cancers [22]. Similarly, the progesterone receptor (PR) exists in two forms, PRA and PRB. These receptors (ERα, PRA, and PRB) are typically identified using immunohistochemical techniques on biopsy tissue samples. A critical threshold for the positive identification of these receptors is their expression greater than or equal to 1% of tumor cells. Notably, the expression of PR often increases as a result of ER signaling; hence, cells expressing ER are likely to express PR as well. It is rare for tumors that are ER-negative to be PR-positive [23,24].

The primary clinical significance of hormone receptors lies in selecting patients for adjuvant therapy with hormonal drugs. These treatments include selective ER modulators (such as tamoxifen), third-generation aromatase inhibitors (like anastrozole, letrozole, or exemestane), LH-RH agonists (including leuprolide and goserelin), pure ER antagonists (such as fulvestrant), oophorectomy, or other endocrine therapies [25]. Consequently, these receptors have predictive utility. Additionally, they offer prognostic utility; hormone receptor-positive tumors have been linked to improved survival and a lower annual recurrence rate within the first five years post treatment [26].

Conversely, HER2, another critical biomarker, belongs to the epidermal growth factor receptor (EGFR) family and plays a role in numerous tumor signaling pathways. Its analysis is generally conducted on biopsy tissue samples, utilizing immunohistochemistry or in situ hybridization techniques. Luminal-type breast cancers, characterized by low HER2 receptor expression, are typically not considered for anti-HER2 treatments like trastuzumab or pertuzumab [27].

Additionally, the Ki-67 protein serves as an important biomarker for assessing tumor proliferative activity, given its involvement in cell division. Although there is no universally established cutoff point, major guidelines suggest that high proliferative activity is indicated by values above 30%, while low activity is below 10% [28,29]. For instance, a Ki-67 level of 20% indicates that 20% of the tumor cells are actively dividing. Generally, luminal tumors exhibit low Ki-67 levels. In research conducted by Viale et al., the prognostic and predictive value of Ki-67 was explored, revealing that higher values correlate with a poorer prognosis [30].

### 2.2. Serological Biomarkers

Serological markers are of significant importance in both the diagnosis and prognosis of breast cancer, with Ki67, CA 15-3, BAX, and Bcl-2 being notably characteristic in luminal subtype tumors [31,32]. Although Ki-67 is typically assessed in tumor tissue, its potential as a serological biomarker for estimating cell proliferation has been explored. For instance, a study by Cheang et al. revealed that Luminal B tumors exhibited a higher Ki67 index and were associated with poorer recurrence-free survival compared to Luminal A tumors [33].

Focusing on the role of BAX and Bcl-2, these proteins are intricately linked to apoptosis and their expression carries prognostic implications in breast cancer. BAX is crucial in inducing apoptosis, whereas Bcl-2 prevents programmed cell death. This balance is key to cell survival. A specific study examining the expression of Bcl-2 and BAX as prognostic markers in breast cancer found that Bcl-2 expression correlated with a better prognosis across all molecular subtypes, including Luminal A breast cancer [34,35].

Another important serological marker is CA 15-3, a protein component of MUC 1 found in epithelial cells. It is frequently utilized as a tumor marker for detecting and monitoring breast cancer [36]. However, it is noteworthy that CA 15-3 levels can also rise in other conditions, such as gastrointestinal and lung neoplasms. The specificity of CA 15-3 as a prognostic marker for Luminal A breast cancer requires further study. Additionally, carcinoembryonic antigen (CEA) and CA 27.29 are other tumor markers relevant in breast cancer, especially for monitoring patients in advanced stages. Elevated CEA levels may signal tumor activity but can also increase due to non-cancerous conditions like inflammatory gastrointestinal diseases. CA 27.29 may be particularly valuable in metastatic breast cancer [37].

Moreover, chronic inflammation, with cytokines acting as mediators, is recognized as a risk factor for tumor development. Research involving the detection of cytokines in the blood for tumor diagnosis has shown preliminary utility. Cytokines such as CXCL12, CXCL1, CXCL8, and CXCR4, when combined with the CA 15-3 antigen panel, may serve as early biomarkers in the diagnosis of breast cancer, particularly for luminal-type cancers [38].

### 2.3. Genetic Biomarkers

It is crucial to acknowledge that the identification of genetic alterations in breast cancer is essential not only for determining prognosis and guiding treatment decisions but also for genetic counseling and screening of individuals at risk. Gene expression analysis platforms, such as Oncotype DX and MammaPrint, analyze multiple genes from tumor tissue samples. These analyses provide insights into the tumor’s specific characteristics, including its aggressiveness, recurrence risk, and the necessity for therapy initiation [39]. Within this framework, genetic alterations in breast cancer can be broadly categorized into two types: somatic mutations and germline mutations.

Somatic mutations, which are unique to tumor cells and not passed down through generations, play a significant role in breast cancer. In Luminal A breast cancer, a primary somatic mutation is found in the PIK3CA gene in approximately 49% of cases [40]. This gene is instrumental in cell signaling regulation and is a part of the PI3K/AKT/mTOR pathway, often leading to enhanced cell growth and survival [41]. Another noteworthy gene is GATA3, which is involved in cellular differentiation and is present in 14% of Luminal A tumors. GATA3 expression is associated with higher hormonal receptor expression and a more favorable prognosis [42]. Additionally, the TP53 gene, known for its critical role in maintaining DNA integrity, is altered in about 12% of cases, often indicating increased tumor aggressiveness and a poorer prognosis [43]. The MAP3K1 gene, with a mutation rate of 14% in this tumor subtype, highlights the genetic heterogeneity of luminal tumors. However, its exact role remains to be fully understood. It has been observed that specific genes, like MCM4, correlate with survival in the Luminal A subtype but not in Luminal B, HER2-positive, or triple-negative subtypes [44].

In contrast, germline mutations are inherited from one’s parents and are present in all body cells, including germ cells. A well-studied genetic alteration in breast cancer is the BRCA1 gene mutation. Individuals with this mutation have an elevated risk of developing breast cancer, particularly at a younger age [45]. These mutations are more prevalent in hereditary breast cancer cases and are linked to a higher likelihood of developing bilateral breast cancer and luminal-type tumors. Awareness of BRCA1 mutations can significantly influence treatment choices, such as the consideration of prophylactic mastectomy [46]. However, it is important to note that while germline mutations increase the risk of breast cancer, they do not definitively determine whether an individual will develop the disease [47].

### 2.4. Circulating Tumor Cells and MicroRNAs

MicroRNAs (miRNAs) are small RNA molecules playing a crucial role in gene expression regulation. Acting as either oncogenes or tumor suppressors, their impact is determined by their specific functions and the genes they regulate. The exploration of miRNA expression abnormalities in breast cancer, including the Luminal A subtype, represents a significant area of contemporary research. This research is key to understanding the molecular architecture of these tumor types [48].

A prominent miRNA in Luminal A breast cancer is miR-21. Research, including a study by Kalinina et al., indicates that miR-21 overexpression may foster tumor cell proliferation and hinder apoptosis. Furthermore, there is evidence suggesting that miR-21 might influence the response to hormonal therapy, a cornerstone in the treatment of Luminal A breast cancer [49]. Additional miRNAs associated with Luminal A breast cancer include miR-34a, miR-126, miR-206, and the miR-221/222 cluster [50]. These miRNAs are being closely studied for their roles in the progression and treatment response of this cancer subtype.

The investigation of circulating tumor cells (CTCs) in the peripheral blood of patients with non-metastatic breast cancer is increasingly being recognized as crucial in both research and clinical practice. CTCs, which are tumor cells that break away from the primary tumor and enter the bloodstream, possess the ability to migrate to distant sites and potentially initiate metastases. Their detection is typically conducted using methods like flow cytometry, polymerase chain reaction (PCR), and various cell capture techniques. A notable correlation has been established between the presence of CTCs and poorer clinical outcomes, including reduced progression-free and overall survival. This association underscores the potential of CTCs as valuable prognostic indicators in early-stage breast cancer [51].

Recent advancements in research suggest that the identification and analysis of CTCs could significantly enhance risk stratification and the personalization of treatment for breast cancer patients. Particularly in non-metastatic cases, analyzing CTCs can offer insights into minimal residual disease—the presence of cancer cells remaining in the body post initial treatment. This information is vital for assessing the efficacy of adjuvant therapies, thereby aiding in customizing treatment plans to optimize outcomes and monitoring [52]. Pierga et al.’s research indicated that the detection of CTCs can predict early metastatic recurrence following neoadjuvant chemotherapy in operable and locally advanced breast cancer patients [53]. Furthermore, a study examining the expression of epidermal growth factor receptor (EGFR) and the cell surface protein CD133 in CTCs of breast cancer patients found a significant link between positive EGFR expression in CTCs and luminal-type tumors [54].

In conclusion, understanding various biomarkers is crucial for personalized medicine in Luminal A breast cancer. Histological biomarkers, including hormone receptors (ERα, PRA, PRB), HER2, and Ki-67, are key in diagnosis, prognosis, and treatment. Serological markers like Ki-67, CA 15-3, BAX, and Bcl-2 are important for prognosis and monitoring. Genetic biomarkers, both somatic (e.g., PIK3CA, GATA3, TP53, MAP3K1 mutations) and germline (like BRCA1 mutations), provide insights into the disease’s origins and treatments. MicroRNAs, especially miR-21, play a role in tumor biology and response to therapy. Circulating tumor cells (CTCs) have emerged as a novel biomarker for understanding metastasis and prognosis in non-metastatic breast cancer, helping in assessing residual disease and treatment planning.

## 3. Luminal B

Luminal breast cancer accounts for approximately two-thirds of all breast carcinomas globally. Presently, there is an emphasis on individualized therapies tailored to the biological characteristics unique to each luminal subtype. Within these subtypes, Luminal B breast cancer is comparatively less common. Similar to Luminal A, Luminal B is characterized by positive hormone receptors. However, in contrast to Luminal A, which is typically HER2-negative, Luminal B can be either HER2-positive or -negative. Notably, Luminal B is associated with a higher rate of cell proliferation than Luminal A, which correlates with a less favorable prognosis due to its increased aggressiveness. In the context of early-stage breast cancer, Luminal B demonstrates poorer outcomes in terms of 5- and 10-year event-free survival (EFS), irrespective of adjuvant systemic therapy, when compared to Luminal A [55].

### 3.1. Histological Biomarkers

As previously mentioned in the context of Luminal A breast cancer, Luminal B tumors are also characterized by a high expression of ERs and PRs. This characteristic renders these tumors responsive to endocrine therapy targeting these receptors, often leading to a better prognosis due to the availability of an effective therapeutic target. However, despite the general effectiveness of hormonal therapy, some patients with Luminal B breast cancer may develop resistance over time. This resistance can manifest as cancer recurrence or continued tumor growth despite ongoing hormonal therapy [56]. The mechanisms behind hormone resistance are varied, encompassing alterations in hormone receptor expression, engagement of alternative signaling pathways, and genetic modifications.

A critical aspect of Luminal B breast cancer is the expression of the HER2 receptor, which significantly influences both treatment and prognosis. Contrary to Luminal A, which is usually HER2-negative, Luminal B can exhibit HER2 positivity. Recent updates in the classification of intrinsic subtypes now include Luminal B with HER2 overexpression [57]. This distinction is crucial for treatment considerations; while HER2 positivity may worsen the prognosis, it also opens the possibility for targeted therapies against HER2, such as trastuzumab. In cases where HER2 is not overexpressed, the treatment strategy primarily revolves around hormonal therapy and chemotherapy, tailored to the individual patient’s characteristics [58].

The Ki-67 index, a key marker for Luminal B breast cancer, can be evaluated using immunohistochemical techniques on tissue samples. Luminal B is distinguished by a higher Ki-67 percentage than Luminal A, correlating with a less favorable prognosis, an increased risk of recurrence, and faster disease progression [59]. Ki-67 levels also play a significant role in determining treatment approaches. Patients with Luminal B breast cancer exhibiting high Ki-67 levels are often considered for adjuvant chemotherapy or a combination of a cell cycle inhibitor with hormonal therapy [60]. Cheang et al. developed an immunohistochemical assay to differentiate Luminal B tumors from Luminal A based on the Ki-67 index and explored its utility in predicting breast cancer recurrence-free survival and overall disease survival [61].

### 3.2. Serological Biomarkers

Numerous serological markers have been identified as crucial in determining prognosis, aiding in treatment selection, and understanding the behavior of Luminal B breast cancer. Beyond the Ki-67 index, which shows elevated expression in both tissue and blood in Luminal B tumors, indicating a poorer prognosis compared to Luminal A, there are other cell proliferation markers of interest. These include the proliferating cell nuclear antigen (PCNA) and topoisomerase II, which may play significant roles in the pathology of Luminal B breast cancer [62].

Additionally, the expression of specific genes and proteins such as p62, also known as sequestosome-1 (SQSTM1), has been identified. p62 is instrumental in the regulation of autophagy and selective protein degradation in cells. Another notable marker is aldehyde dehydrogenase 1 family member A3 (ALDH1A3), involved in aldehyde oxidation, potentially influencing chemotherapy resistance. The presence of these markers is associated with a poorer prognosis in Luminal B breast cancer [63].

Furthermore, components of the MCM complex, including MCM2, MCM4, and MCM6, are essential proteins involved in DNA replication. These proteins are vital in the preparation and initiation of DNA replication, a key process for cell proliferation. In differentiating between breast cancer subtypes, MCM2, MCM4, and MCM6 gain relevance due to their association with cell proliferation. Given that Luminal B breast cancer is characterized by a higher rate of cell proliferation compared to Luminal A, the expression levels of these markers may be elevated in Luminal B, thus aiding in the distinction between these subtypes [64].

### 3.3. Genetic Biomarkers

Luminal B breast cancer is characterized by several significant genetic alterations with varying frequencies. Beyond the expression of hormonal receptors, this subtype may also exhibit an overexpression of HER-2 in some cases, which is linked to a poorer prognosis [65,66,67]. Research has indicated that certain genetic polymorphisms are associated with the Luminal B subtype, suggesting their potential in predicting subtypes and guiding personalized treatment strategies [68,69].

Mutations in the PIK3CA gene, present in about 32% of Luminal B cases, can activate the PI3K/AKT/mTOR signaling pathway, contributing to breast cancer development and progression. Additionally, TP53 gene mutations are relatively common in this subtype, occurring in approximately 31% of cases, which is a higher frequency than in Luminal A breast cancer [70]. These mutations are considered possible predictors of resistance to endocrine therapy. Both TP53 and PIK3CA mutations are associated with increased tumor aggressiveness and an unfavorable prognosis [71].

In response to these genetic alterations, targeted therapies have been developed, such as PI3K inhibitors like alpelisib and idelalisib, and mTOR inhibitors like everolimus. These therapies are selected based on tumor characteristics and patient response to treatment [72]. These genes are instrumental in the proliferation and prognosis of Luminal B breast cancer, providing potential targets for diagnosis, prognosis, and treatment strategies. Additionally, in the metastatic setting, other less common biomarkers for targeted therapy options (MSI/MMR, TMB, NTRK) and comprehensive profiling of genomic profiles are utilized to identify uncommon targets and determine additional treatment options.

Moreover, research into non-coding cis-regulatory elements, DNA regions that do not encode proteins but are crucial in gene expression regulation, has highlighted their potential impact on gene activity. These elements contribute to the molecular and biological characteristics of Luminal B breast cancer, offering insights into the subtype’s distinct nature [73].

### 3.4. Circulating Tumor Cells and MicroRNAs

In Luminal B breast cancer, the influence of microRNAs on disease progression and treatment response has been a subject of considerable research. One microRNA that has been extensively studied in this context, similar to its role in Luminal A, is miR-21. This microRNA is linked with tumor proliferation and resistance to chemotherapy in breast cancer. Song et al.’s research revealed that 25 out of 32 histological breast tissue samples from cancer patients showed miR-21 overexpression compared to the normal mammary epithelium. Furthermore, a close correlation was observed between the incidence of lymph node metastasis and miR-21 expression, suggesting its significant role in metastasis and, therefore, in prognostication. The study also found that four different breast cancer cell lines exhibited varying levels of miR-21 overexpression, hinting at its potential as a classifier for these tumors [74].

Another microRNA, miR-145, has been recognized as a potential tumor suppressor in breast cancer. Research indicates that miR-145 can inhibit angiogenesis and tumor growth by suppressing N-RAS and VEGF [75]. It is also known to hinder breast cancer cell migration by regulating TGF-β1 expression, either directly or indirectly, further underscoring its tumor-suppressive capabilities [76]. Additionally, microRNAs like miR-221 and miR-222 have been associated with a decreased expression of hormonal receptors and a diminished response to hormonal treatments, highlighting their importance in the context of Luminal B breast cancer [77].

In Luminal B breast cancer, circulating tumor cells (CTCs) can express specific markers such as hormone receptors (ERs/PRs) and HER2, which are distinctive features of this breast cancer subtype. The expression of these markers in CTCs can vary, providing insights into tumor heterogeneity and its evolution during disease progression [78]. Although detecting CTCs poses challenges due to their low concentration in blood, their prognostic significance has been established by various studies. However, as of now, there is no definitive evidence supporting the use of any biomarker for stratifying patients based on individual prognosis or for guiding personalized treatment in this context [79].

A study by Galardi et al. explored the prognostic role of CTC count and its utility in monitoring treatment with palbociclib, a kinase inhibitor, as well as in predicting treatment response. This study concluded that initial CTC count did not provide a clear predictive value in patients treated with palbociclib. However, CTCs proved useful as indicators for identifying patients who were developing early resistance to the treatment. Additionally, CTC counts at the time of disease progression were shown to offer valuable prognostic information regarding subsequent responses to palbociclib [80].

The quantity of detected CTCs has been linked with the prognosis of breast cancer patients. These cells can also offer information about treatment response and the effectiveness of therapies. The field of CTC research in breast cancer is dynamic and continually evolving, with new technologies and methodologies being developed to improve the sensitivity and specificity of CTC detection.

**Figure 1:**
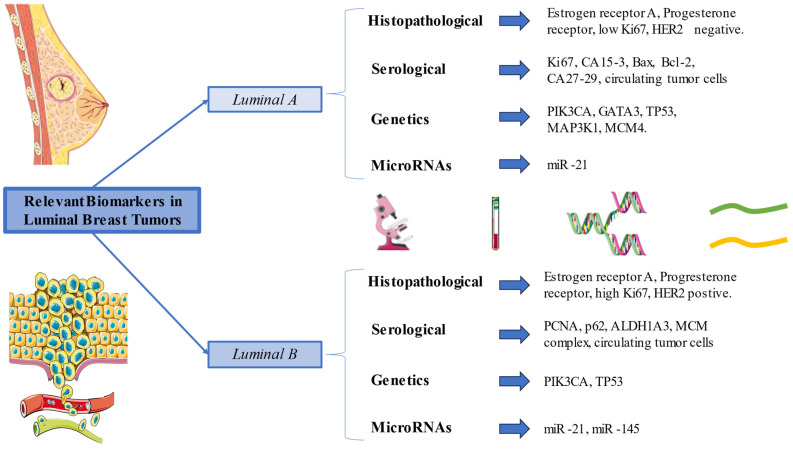
Summary of the most relevant biomarkers in metastatic Luminal A and B tumors.

In summary, Luminal B breast cancer, distinguished by its aggressiveness, shares some markers with Luminal A but crucially differs in HER2 positivity and a higher Ki-67 index, signaling a more challenging prognosis. These markers not only guide but also necessitate targeted therapies and often more aggressive treatment like chemotherapy. Furthermore, serological markers and CTC analysis provide deeper insights into tumor characteristics, crucially aiding in the selection of targeted therapies, particularly against the PI3K/AKT/mTOR pathway. Additionally, microRNAs such as miR-21 and miR-145 are instrumental in understanding disease progression. Overall, the integration of various biomarkers is essential for tailoring effective treatments and improving patient outcomes in Luminal B breast cancer. The most relevant histological, serological, and genetic markers, as well as microRNAs of luminal-type metastatic breast cancer, are concisely summarized in Figure 1.

## 4. HER2

### 4.1. Histological Biomarkers

Approximately 30% of breast cancers test positive for human epidermal growth factor receptor 2 (HER2), previously known as HER2/neu or ERBB-2. HER2, a transmembrane receptor with tyrosine kinase activity, belongs to the epidermal growth factor receptor (EGFR) family and plays a crucial role in cell signaling, differentiation, and angiogenesis [81].

Typically, HER2-positive breast cancer does not express hormone receptors like ER or PR and is characterized by a high proliferative index (KI-67). Initially, the overexpression or amplification of HER2 is assessed using immunohistochemistry (IHC), rated on a scale from 0 to 3 based on staining intensity. In ambiguous cases, such as IHC grade 2, in situ hybridization techniques like fluorescence in situ hybridization (FISH) and chromogenic in situ hybridization (CISH) are employed. These methods determine the amplification of specific genes, including HER2, by identifying and quantifying extra gene copies, which are indicative of increased cancer risk and aggressiveness [82]. FISH utilizes fluorescent probes, while CISH employs chromogenic enzyme-marked probes, resulting in a visible color change under the microscope. Both techniques are essential in determining HER2 status and guiding treatment decisions.

The clinical value of HER2 testing lies in its predictive power, identifying women who could benefit from targeted treatments. Therefore, assessing HER2 status in new cases of invasive and metastatic breast cancer is recommended according to various guidelines. The main treatments targeting HER2 include drugs like trastuzumab, pertuzumab, and lapatinib [83]. Conversely, HER2 overexpression has been linked to drug resistance, such as to paclitaxel, and increased tumor aggressiveness [84]. Although HER2 detection has prognostic value, indicating higher recurrence and mortality rates without adjuvant therapy, the clinical significance of this information is debatable, particularly with early use of HER2-directed agents in treatment.

HER2-positive breast cancer displays intertumoral heterogeneity, with up to 45% of cases expressing hormone receptors and exhibiting various molecular subtypes [85]. While hormone receptor status does not define the overall genetic profile, specific genetic aberrations characterize HER2-positive cancer subgroups. HER2-enriched tumors respond well to anti-HER2 therapy, potentially reducing chemotherapy requirements. However, “HER2 low” tumors, with fewer HER2 receptors, respond poorly to HER2-targeted treatments, prompting the exploration of alternatives like immunotherapy [86].

The presence of tumor-infiltrating lymphocytes (TILs) in tissue samples signals the immune response against cancer cells. TIL assessment is a growing research area with implications for prognosis and treatment. Higher TIL quantities often correlate with better outcomes in certain breast cancer subtypes, suggesting that an active immune response can limit tumor growth and progression. Studies indicate that TIL presence, particularly in triple-negative and HER2-positive tumors, may enhance responsiveness to chemotherapy and anti-HER2 treatments [87].

### 4.2. Serological Biomarkers

In HER2-positive breast cancer, monitoring biomarkers in the blood can offer valuable insights into treatment response and disease progression. While HER2 overexpression is commonly analyzed in tissue samples, HER2/neu can also be detected in circulating DNA, which is released by tumor cells into the bloodstream. This detection of HER2 amplification in circulating DNA serves as an indicator of tumor burden and response to treatment [88].

Similarly to other molecular subtypes of breast cancer, tumor markers like CA 15.3, CEA, and CA 27.29 are also useful. These markers aid in diagnosis and help monitor the effectiveness of specific treatments.

### 4.3. Genetic Biomarkers

HER2-positive breast cancer patients often exhibit intratumoral genetic variability, a phenomenon widely observed in various human cancers, including breast cancer. In these cancers, HER2 overexpression and amplification can display a heterogeneous pattern [89]. Three types of cellular distributions have been identified based on HER2 status heterogeneity: “clustered”, featuring two distinct tumor clones (one with HER2 amplification and another with a normal status); “mosaic”, where a diffused mix of cells with different HER2 states exists; and “dispersed”, characterized by isolated HER2-amplified cells amidst a majority of HER2-negative tumor cells.

Genomic analysis, including gene copy number profiling and massive parallel sequencing, has been conducted on heterogeneous HER2 breast cancers. This analysis has identified driver genetic alterations restricted to HER2-negative cells. In vitro models have shown that the overexpression/amplification of BRF2 and DSN1, along with the HER2 I767M mutation, compensate for the lack of HER2 amplification in the HER2-negative components of these breast carcinomas [90].

Studies have linked the increased frequency of chromosome 17 polysomy with HER2 heterogeneity in breast cancer. While rare, chromosome 17 polysomy often appears as a gain or amplification of the centromere of chromosome 17 (CEP17). In these cases, it is crucial to refer to it as “abnormal CEP17 copy number”, particularly when evaluated using FISH in interphase nuclei. The independent increase in CEP17 copy numbers in heterogeneous HER2 carcinomas suggests a possible link to chromosomal instability [91].

The response to neoadjuvant chemotherapy in HER2-positive breast cancer can also vary based on PIK3CA mutations and hormone receptor status. In HER2-positive/ER-positive patients, a better prognosis and reduced benefit from trastuzumab have been reported, along with lower rates of TP53 mutations and reduced HER2 expression [92]. Other mutations in genes like p53, PTEN, PIK2CA, or TOP2A may contribute to the progression and treatment resistance of HER2-positive breast cancer.

Furthermore, mutations in BRCA1 and BRCA2 are more commonly associated with HER2-positive breast cancer. Research suggests that HER2-positive patients with BRCA mutations might be more sensitive to certain treatments, including PARP inhibitors like olaparib [93].

### 4.4. Circulating Tumor Cells and MicroRNAs

The role of microRNAs in HER2-positive breast cancer has garnered significant interest in recent research. Various studies have explored how microRNAs regulate HER-2 and impact the progression of this breast cancer subtype. For instance, microRNAs such as microRNA-125a, microRNA-125b, and microRNA-148a have been identified for their role in regulating HER-2, showing an inhibitory effect on its expression under certain conditions [94].

Similar to its role in luminal tumors, microRNA-21 in HER2-positive breast cancer has been linked to the activation of the HER-2 pathway. It functions by suppressing tumor suppressor genes and promoting cell proliferation, a role paralleled by microRNA-205. Conversely, research suggests that microRNA-26a may inhibit cell proliferation and invasion in HER2-positive breast cancer by modulating the expression of genes associated with these processes [95].

In HER2-positive breast cancer, research has focused on the expression of specific molecules in circulating tumor cells (CTCs). One key area of study is the detection of HER2/Neu mRNA in the blood, which helps assess the presence of the HER2 protein in CTCs. High levels of HER2/Neu mRNA are linked to increased risks of recurrence and mortality [96].

Cytokeratin 19 (CK19), found in epithelial cells, is a common immunohistochemical marker in breast cancer diagnosis. Its presence in cancer cells confirms the epithelial origin of the tumor and aids in identifying specific breast cancer subtypes. CK19’s role extends to evaluating tumor aggressiveness and response to treatments, including HER2-targeted therapies. The presence of CK19 mRNA in CTCs is associated with a worse prognosis, particularly in patients with triple-negative and HER2/Neu-negative tumors, indicating potential aggressiveness and treatment resistance [97,98].

Additionally, the breast-specific protein mammaglobin and its expression in CTCs are being studied in HER2-positive breast cancer. Mammaglobin mRNA in the blood correlates with shorter survival times and lower overall survival rates in these patients [99].

In summary, HER2-positive breast cancer, notable for its HER2 receptor overexpression and lack of hormone receptors, makes up about 30% of breast cancers. HER2 status, key for treatment decisions, is assessed using immunohistochemistry and, if necessary, in situ hybridization techniques like FISH and CISH. The presence of HER2 overexpression in CTCs directs the use of targeted therapies such as trastuzumab or lapatinib. Serological biomarkers like CA 15.3 and CEA are important for monitoring disease progression. This cancer subtype also shows genetic variability, including HER2 heterogeneity and mutations in genes like PIK3CA, which affect treatment response and prognosis. MicroRNAs such as microRNA-125a and -21 also play roles in regulating HER-2 expression and cancer progression. The analysis of molecules in CTCs, like HER2/Neu mRNA, provides insights into recurrence risk and treatment response. Overall, the combination of histological, serological, genetic biomarkers, microRNAs, and CTCs is crucial in understanding HER2-positive breast cancer and developing personalized treatment plans.

## 5. Therapeutic Implication of Specific Biomarkers in Luminal A, Luminal B, and HER-2 Breast Cancer

### 5.1. CDK4/6 Inhibitors

The CCND1 gene is amplified in approximately 15% of breast cancer cells, and cyclin D1 is overexpressed in up to 67%. Overexpression of cyclin D1 increases cyclin-dependent kinases 4 and 6 (CDK4/6) and stimulates cell division, making this pathway an attractive target in cancer therapy [100,101,102,103,104]. Abemaciclib is a small-molecule inhibitor of CDK4, CDK6, and CDK9. Amplification of CCND1 and overexpression of cyclin D1 are common in hormone receptor-positive breast cancers, implying greater sensitivity to CDK4/6 inhibition.

The clinical trial MONARCH 2 [100], which evaluated abemaciclib for advanced HR+/HER2- breast cancer that progressed during or after endocrine therapy, significantly reduced the risk of disease progression or death. The median progression-free survival was 16.4 months with abemaciclib compared to 9.3 months with a placebo. In MONARCH 3 [101], abemaciclib improved progression-free survival in first-line treatment for advanced HR+/HER2- breast cancer in postmenopausal women, in combination with aromatase inhibitors. Abemaciclib was also approved as a second-line treatment based on the results of the MONARCH 1 trial [102]. The monarchE trial [103] demonstrated a clinically significant benefit by reducing invasive disease events when abemaciclib was added to endocrine therapy in high-risk early-stage HR+/HER2- breast cancer.

Palbociclib is another potent CDK4/6 inhibitor, and the PALOMA-2 trial [104] demonstrated its effectiveness in first-line treatment for advanced HR+/HER2- breast cancer in postmenopausal women. The combination of palbociclib and letrozole significantly increased progression-free survival compared to the placebo. The PALOMA-3 trial [105] with palbociclib evaluated its effectiveness in combination with fulvestrant as second-line treatment for advanced HR+/HER2- breast cancer. Palbociclib doubled the median progression-free survival compared to the placebo.

The MONALEESA-2 trial [106] evaluated ribociclib as a first-line treatment for advanced HR+/HER2- breast cancer in postmenopausal women, showing a significant improvement in progression-free survival and overall survival. The MONALEESA-3 trial [107] compared ribociclib as a first- and second-line treatment for advanced HR+/HER2- breast cancer, demonstrating a significant improvement in progression-free survival and overall survival in both settings. MONALEESA-7 [108] investigated ribociclib in combination with endocrine therapy for early-stage HR+/HER2- breast cancer in premenopausal and perimenopausal women, showing a substantial increase in progression-free survival and overall survival.

Despite the clinical success of CDK4/6 inhibitors, they have side effects such as diarrhea, thromboembolism, hematological toxicity, and interstitial lung disease. Resistance to these inhibitors is an ongoing challenge, and studies like neoMONARCH [109] explore molecular signatures associated with sensitivity or resistance to guide treatment decisions.

In conclusion, amplification of CCND1 and overexpression of cyclin D1 are key in many breast cancers, making CDK4/6 inhibitors like abemaciclib, palbociclib, and ribociclib effective treatments. Clinical trials have confirmed their benefits in various stages of breast cancer, though challenges remain with side effects and resistance. Ongoing research is crucial to enhance treatment efficacy and patient response.

### 5.2. mTOR Inhibitors

The mammalian target of rapamycin (mTOR) is a protein kinase that plays a role in cellular proliferation and survival. Rapamycin, also known as sirolimus, inhibits mTOR signaling by binding to the FK-binding protein 12 (FKBP12), disrupting the mTOR Complex 1 (mTORC1). Rapamycin analogs (rapalogs) were developed to enhance solubility and pharmacokinetic properties.

Everolimus, an mTOR inhibitor, was evaluated in the BOLERO-1 trial [110] as a first-line treatment for advanced HER2+ breast cancer. When combined with trastuzumab and paclitaxel, everolimus did not show improvements in key outcomes in the overall population but did benefit HR-/HER2+ patients. The interaction between the HR, HER2, and PI3K pathways could contribute to resistance in HR+/HER2+ patients.

The combination of everolimus with letrozole synergistically inhibited cell proliferation and induced apoptosis in ER+ breast cancer cells. The BOLERO-2 trial [111] studied everolimus with exemestane in non-steroidal aromatase inhibitor refractory breast cancer, showing an increase in progression-free survival. Everolimus, in combination with exemestane, was approved in 2012 for advanced HR+/HER2- breast cancer. mTOR inhibitors, such as everolimus, have various adverse effects, including neutropenia, stomatitis, diarrhea, and alopecia. Ongoing research explores their potential in triple-negative breast cancer (TNBC), where preclinical studies suggest a cytostatic effect [112].

To summarize, mTOR inhibitors like everolimus have shown promise in treating advanced breast cancer. While results in HER2+ cases were mixed, they have been effective in HR+ breast cancer, especially when combined with drugs like letrozole and exemestane. Approved for advanced HR+/HER2- breast cancer in 2012, these inhibitors, despite their side effects, are now being explored for potential use in triple-negative breast cancer.

### 5.3. PI3K Inhibitors

Approximately 40% of HR+/HER2- breast cancers have activating mutations in the PIK3CA gene, leading to the hyperactivation of the catalytic subunit p110α of phosphatidylinositol 3-kinase (PI3K). Mutations in PI3K p110α promote tumor-like behavior in mammary epithelial cells and confer resistance to endocrine therapy and conventional chemotherapy, correlating with unfavorable clinical outcomes.

The clinical trial SOLAR-1 [113] enrolled postmenopausal patients with advanced HR+/HER2- breast cancer who experienced disease progression during or after aromatase inhibitor therapy. Participants were administered alpelisib, a PI3K inhibitor, or a placebo in combination with the estrogen receptor antagonist fulvestrant. A modest clinical benefit was observed in the non-mutated PIK3CA subgroup, with alpelisib increasing progression-free survival by 1.8 months, although the effect was not statistically significant. However, for patients with PIK3CA mutations, the median progression-free survival almost doubled, from 5.7 months with placebo to 11.0 months with alpelisib. Based on the positive results of SOLAR-1, the FDA approved alpelisib in combination with fulvestrant for advanced HR+/HER2- breast cancer with PIK3CA mutations in postmenopausal women.

The most common grade 3 or higher adverse events with alpelisib are hyperglycemia and maculopapular rash. Both are considered specific effects of PI3Kα inhibition, owing to the role of this pathway in glucose metabolism and the differentiation and survival of keratinocytes. Indeed, there have been several case reports of alpelisib-induced diabetic ketoacidosis, even in non-diabetic patients.

The utility of alpelisib as a second-line agent is complicated by the recent availability of CDK 4/6 inhibitors and their introduction into the standard first-line treatment for HR+/HER2- breast cancer. In the SOLAR-1 trial, only nine patients (5%) who received alpelisib had previously been treated with a CDK 4/6 inhibitor. BYLieve is a non-comparative Phase II trial of alpelisib plus endocrine therapy [114]. However, larger comparative studies are needed to better define the role of alpelisib in breast cancer therapy. Ongoing Phase III clinical trials explore the use of alpelisib with other agents, including its combination with trastuzumab and pertuzumab for HER2+ breast cancer with PI3KCA mutations (EPIK-B2) [115] and with nab-paclitaxel for triple-negative breast cancer (EPIK-B3) [116]. The results of these and other trials are expected to better delineate the utility of alpelisib and possibly define new roles for its application in the therapy of metastatic disease.

In brief, alpelisib, a PI3K inhibitor, has proven effective for HR+/HER2- breast cancer with PIK3CA mutations, as shown in the SOLAR-1 trial. It notably increases progression-free survival and is FDA-approved in combination with fulvestrant. Despite its efficacy, alpelisib’s role in treatment regimens is evolving, particularly due to side effects like hyperglycemia, and ongoing research is comparing it with other therapies.

### 5.4. Anti-HER2 Antibodies

HER2 is a transmembrane receptor tyrosine kinase involved in various cellular processes. The amplification of the HER2 gene is linked to poor outcomes in breast cancer, leading to the development of agents targeting HER2. Trastuzumab, an anti-HER2 monoclonal antibody, was the first drug developed and approved for clinical use. It demonstrated efficacy in metastatic and early-stage HER2-positive breast cancer, showing benefits in terms of progression-free survival and overall survival. There are clinical trials, such as H0648g, M77001, BCIRG-006, and HERA, which assess the efficacy of trastuzumab in different settings, including metastatic and adjuvant treatment [117]. Although these drugs have significantly improved outcomes, they can also cause adverse effects, such as cardiotoxicity and pulmonary toxicity.

Pertuzumab, an anti-HER2 drug that acts on a different domain of the receptor than trastuzumab, showed synergy when combined with trastuzumab and chemotherapy. Clinical trials such as CLEOPATRA [118] and APHINITY [119] demonstrated the benefits of dual HER2 blockade in both metastatic breast cancer and early-stage high-risk settings. Its effectiveness has been demonstrated in patients with affected lymph nodes and small tumors. Although there are therapeutic issues that still need clarification, such as early recurrence in HER2+ breast cancer, anti-HER2 monoclonal antibodies are also essential in advanced disease. The combination of trastuzumab–pertuzumab with chemotherapy is standard in first-line treatment, and subsequent lines of treatment largely depend on these antibodies, often in combination with other HER2-targeted drugs, such as tyrosine kinase inhibitors. One advantage is that subcutaneous formulations of anti-HER2 antibodies with hyaluronidase have been developed and have been approved by the Food and Drug Administration, providing an alternative to the intravenous route [120].

Margetuximab is a monoclonal antibody against HER2 that, compared to trastuzumab, features a modified Fc domain, increasing affinity for CD16A and reducing affinity for CD32B. This modification enhances antibody-dependent cellular cytotoxicity (ADCC) of the innate immune system. In the SOPHIA trial [121], which compared margetuximab with trastuzumab in the treatment of advanced breast cancer following progression after HER2-targeted therapies, margetuximab demonstrated an increase in progression-free survival by approximately 1 month in the intention-to-treat population. The benefit of margetuximab was selective for patients with the CD16A-158F allele, while it did not provide clinical benefit in V/V homozygotes CD16A-158 compared to trastuzumab. Based on these findings, the Food and Drug Administration approved margetuximab in combination with chemotherapy as a third-line treatment for metastatic HER2+ breast cancer. Common adverse events were similar to those of trastuzumab, although infusion-related reactions were more frequent with margetuximab.

Several studies have also been conducted on the use of these therapies in neoadjuvant treatment. The Phase II clinical trial, NeoSphere [122], evaluated various combinations of pertuzumab, trastuzumab, and docetaxel in the neoadjuvant setting for HER2+ breast cancer treatment. Although the combination of all three agents showed the highest rate of pathological complete responses (46%), it did not demonstrate superiority in progression-free survival, possibly due to the small size of the study. Another trial, WSG-ADAPT HR-/HER2+ [123], tested the combination of pertuzumab–trastuzumab with or without paclitaxel in the same setting, showing a high rate of pathological complete responses (91%) with the addition of paclitaxel. At 5 years of follow-up, disease-free survival was 98% with paclitaxel and 89% without paclitaxel, although it did not reach statistical significance. Larger trials are needed to assess the significance of survival outcomes, but the high rate of pathological complete responses justifies reconsidering the use of this combination in neoadjuvant treatment.

Therefore, despite lingering therapeutic questions, these anti-HER2 monoclonal antibodies play a crucial role in various stages of HER2+ breast cancer treatment. They are essential tools, demonstrating effectiveness in both adjuvant therapy and advanced disease.

### 5.5. PARP Inhibitors

Mutations in the BRCA1 and BRCA2 genes increase the risk of breast cancer, with cumulative risks of 72% for BRCA1 and 69% for BRCA2 mutation carriers. The use of PARP inhibitors, such as olaparib and talazoparib, has shown effectiveness in treating breast cancer with BRCA1 or BRCA2 mutations. These inhibitors target Poly (ADP-ribose) polymerase (PARP) 1 and 2, crucial in DNA repair. Studies in 2005 demonstrated that tumors with BRCA mutations are particularly sensitive to PARP inhibition, leading to chromosomal instability and apoptosis [124].

The OlympiAD trial [124] compared the efficacy of olaparib with the physician’s choice of single-agent chemotherapy in patients with HER2-negative metastatic breast cancer and germline mutations in BRCA1 or BRCA2 (gBRCAm). Olaparib demonstrated a significant extension in progression-free survival compared to TPC, reducing the risk of disease progression or death by 42% in the intention-to-treat population. Although there was no significant improvement in overall survival in the total population, subgroup analysis revealed a clinically significant benefit in patients without prior treatment. In 2018, the FDA approved olaparib for the treatment of HER2-negative metastatic breast cancer with gBRCAm.

Talazoparib, another PARP inhibitor, was approved by the Food and Drug Administration for use in gBRCAm HER2-negative locally advanced or metastatic breast cancer based on the outcomes of the EMBRACA trial [125], a Phase III trial that compared the efficacy of talazoparib with standard chemotherapy in this subgroup of patients.

To summarize, mutations in BRCA1 and BRCA2 significantly increase breast cancer risk, and PARP inhibitors like olaparib and talazoparib have been effective in targeting these mutations. These inhibitors work by disrupting DNA repair processes, particularly in tumors with BRCA mutations. The OlympiAD trial showed that olaparib notably extended progression-free survival in patients with HER2-negative metastatic breast cancer and BRCA mutations, leading to its FDA approval in 2018. Similarly, talazoparib, proven effective in the EMBRACA trial, was also FDA-approved for treating advanced breast cancer in patients with BRCA mutations. These developments mark significant advancements in personalized breast cancer therapy.

The complexities, clinical trial outcomes, side effects, and ongoing challenges associated with each of these therapeutic agents are summarized in Table 1.

## 6. Triple-Negative

Triple-negative breast cancer (TNBC), characterized by the absence of estrogen receptor (ER), progesterone receptor (PR), and human epidermal growth factor receptor 2 (HER2), accounts for 10–15% of all breast cancers. TNBC’s epidemiologic profile is complex, with notable implications. Epidemiologically, it predominantly affects younger women, making age a significant risk factor. Women under 40 years of age are more likely to be diagnosed with TNBC compared to other breast cancer subtypes.

Beyond age, TNBC shows racial disparities in incidence, disproportionately affecting Hispanic and African American women. These ethnic groups often present with more advanced stages of the disease at diagnosis. Genetics also play a crucial role in TNBC, with a significant number of cases linked to BRCA1 mutations, many of which are hereditary. Understanding the genetic basis of TNBC is essential for effective risk assessment and personalized treatment strategies.

Given TNBC’s aggressive nature and the scarcity of targeted treatments, developing clinical biomarkers for this cancer type is crucial. These biomarkers aim to provide comprehensive information about prognosis, predict treatment response, analyze unique genetic and molecular characteristics for personalized medicine, and identify potential therapeutic targets. Consequently, several serologic, histologic, and genetic biomarkers for TNBC are currently being developed.

### 6.1. Histological Biomarkers: PD-L1 and Novel Histological Biomarkers

Identifying biomarkers that can predict prognosis and guide treatment decisions is critical for improving outcomes in triple-negative breast cancer (TNBC) patients. Recent research has emphasized the clinical importance of programmed death-ligand 1 (PD-L1) in TNBC. PD-L1 has become a significant biomarker in metastatic TNBC, with studies exploring its expression and clinical implications.

One notable finding is PD-L1’s role as a negative prognostic factor in TNBC. Muenst et al. [126] demonstrated that patients with PD-L1-positive tumors had poorer overall and disease-free survival compared to those with PD-L1-negative tumors, suggesting its potential as a prognostic marker. Furthermore, PD-L1 expression in TNBC is crucial, particularly regarding immunotherapy. PD-L1 on tumor cells helps evade immune detection by interacting with the PD-1 receptor on immune cells. Blocking this interaction with immune checkpoint inhibitors, like anti-PD-1 or anti-PD-L1 antibodies, can reactivate antitumor immune responses. The IMpassion130 trial [127] showed that adding the PD-L1 inhibitor atezolizumab to nab-paclitaxel improved survival in patients with newly diagnosed metastatic or locally advanced PD-L1-positive TNBC. The FDA has approved atezolizumab combined with nab-paclitaxel for treating unresectable locally advanced or metastatic PD-L1-positive TNBC [128], representing a new approach in TNBC treatment.

However, challenges remain, including variable PD-L1 testing concordance among pathologists and the dynamic expression of PD-L1. Standardizing PD-L1 testing is essential for accurate clinical decision-making [129]. Immunotherapy targeting PD-L1 shows promise in managing metastatic TNBC, with PD-L1 expression emerging as a predictive indicator for immunotherapy response. While combination treatments have shown enhanced results, further research is needed to understand the response and resistance mechanisms better.

Additionally, tumor-infiltrating lymphocytes (TILs) have gained attention as innovative histological biomarkers in TNBC. Studies, including those by Loi et al. [130] and Adams et al. [131], indicate that a higher quantity of TILs, particularly CD8+ TILs, correlates with improved clinical outcomes in TNBC. This suggests TILs’ potential as a positive prognostic factor. However, caution is needed when assessing TILs using machine learning algorithms, as challenges and limitations exist in this approach [132].

In summary, both PD-L1 and TILs have emerged as important biomarkers in TNBC, offering insights into prognosis and guiding treatment decisions. While PD-L1-targeted immunotherapy has shown promising results, TILs provide a valuable prognostic perspective. Continued research and standardization of assessments are necessary to optimize these biomarkers’ clinical utility in TNBC.

### 6.2. Serological Biomarkers: The Importance of Follow-Up

Serological biomarkers are essential in diagnosing, prognosticating, and managing triple-negative breast cancer (TNBC), a subtype of breast cancer characterized by the absence of estrogen receptor (ER), progesterone receptor (PR), and human epidermal growth factor receptor 2 (HER2) expression. Understanding these biomarkers can provide insights into TNBC and inform personalized treatment approaches.

CA15-3 has emerged as a significant biomarker in TNBC, linked to disease progression and prognosis. Li et al.’s meta-analysis of 36 studies indicated that CA15-3 levels differ among breast cancer subtypes, suggesting its utility in differentiating TNBC from other forms [133]. Fu and Li emphasized the importance of combining CA15-3 with other tumor markers for more accurate clinical assessments [134], while Zhu et al. highlighted its role in monitoring therapy outcomes and disease progression in metastatic breast cancer [135]. Additionally, Wang et al. identified CA15-3 as a key biomarker in nipple discharge, aiding in diagnosis and prognosis [136], and Oliveira et al.’s development of a microfluidic device for CA15-3 detection underscores its practical application in clinical settings [137].

Carcinoembryonic antigen (CEA) is another crucial serological biomarker for monitoring TNBC. Anoop et al. found that elevated serum CEA levels in metastatic breast cancer patients were significantly associated with poorer survival outcomes, indicating its prognostic value in metastatic TNBC [138]. Li et al.’s meta-analysis further supported CEA’s association with larger tumor size, lymph node involvement, and advanced tumor stage [139]. Yang et al.’s study demonstrated that increased CEA levels during therapy could predict a poor therapeutic response [140]. However, CEA is not exclusive to breast cancer and can be elevated in other conditions, necessitating its interpretation alongside other clinical factors.

In conclusion, serological biomarkers like CA15-3 and CEA are promising tools in TNBC management. They offer non-invasive methods for early diagnosis and tailoring treatments. Despite challenges such as validation and standardization, these biomarkers hold the potential to revolutionize the approach and management of this aggressive cancer subtype.

### 6.3. Genetic Biomarkers

Genetic biomarkers are pivotal in understanding the biology of triple-negative breast cancer (TNBC), guiding treatment strategies, and improving patient outcomes. Identifying these biomarkers can aid in diagnosing and prognosing TNBC.

TP53, also known as tumor protein p53, is a tumor suppressor gene crucial for genomic stability. In TNBC, TP53 mutations occur more frequently than in other breast cancer subtypes, associated with a more aggressive phenotype, higher tumor grade, and poorer prognosis [141]. Studies have demonstrated the link between TP53 mutations and higher risks of distant recurrence and reduced overall survival in TNBC patients [142], indicating its prognostic potential. For example, Petrovic et al. found that TP53 mutations were more common in TNBC than other breast cancer subtypes [143]. Moreover, TP53 mutations have been linked to a higher risk of developing TNBC, larger tumor size, and lymph node involvement [144,145]. These mutations also correlate with resistance to common chemotherapeutic agents like anthracyclines and taxanes [146], highlighting their role in guiding treatment decisions.

BRCA1 and BRCA2 mutations are also crucial in TNBC. These tumor suppressor genes are involved in DNA repair, and mutations in them increase breast cancer risk, including TNBC. BRCA mutations are more prevalent in TNBC patients and may offer benefits from targeted therapies like PARP inhibitors. The presence of BRCA mutations necessitates risk assessment and genetic counseling for patients and their families [147]. A study found that testing for BRCA mutations in TNBC patients under 50 could be a cost-effective strategy, reducing the incidence of future breast and ovarian cancers [148]. Noh et al. reported associations between BRCA mutations and younger onset age, higher nuclear grade, and poorer histological grade in TNBC patients [149]. Additionally, these mutations have been linked to increased risks of distant metastasis and decreased survival [150].

TNBC patients with BRCA mutations can benefit from PARP inhibitors, which exploit DNA repair deficiencies caused by these mutations. Clinical trials have shown promising results in terms of response rates and progression-free survival with PARP inhibitor treatments in TNBC patients with BRCA mutations [151,152].

In conclusion, TP53 and BRCA mutations significantly impact the development, progression, and treatment of TNBC. These mutations are associated with increased TNBC risks, specific clinicopathological characteristics, and responsiveness to targeted therapies.

### 6.4. Circulating Tumor Cells and MicroRNAs: New Findings

MicroRNAs (miRNAs) are emerging as key regulators in the pathogenesis of TNBC with their dysregulation implicated in the disease’s progression, diagnosis, and prognosis [153]. Studies have identified specific miRNAs, including miR-145, miR-296, and miR-93, as potential diagnostic and prognostic tools in TNBC [154]. Additionally, circulating miRNAs have been proposed as non-invasive biomarkers for TNBC, aiding in disease monitoring [155]. These miRNAs offer potential for early-stage diagnosis, prognosis, and prediction of therapeutic response.

Their role in TNBC extends to influencing cancer progression, metastasis, and drug resistance [156]. MiRNAs are also involved in regulating the epithelial–mesenchymal transition (EMT) and cancer stem cell properties, impacting the disease’s phenotype [157]. MiRNA expression profiling is instrumental in identifying specific miRNA signatures for TNBC, with bioinformatic analysis revealing miR-934 as a potential EMT regulator [158]. Integrated analysis of data related to circulating miRNAs can unveil drug resistance mechanisms in TNBC [52], highlighting their therapeutic potential.

Circulating tumor cells (CTCs) also play a crucial role in TNBC. Their presence has been linked to early metastatic relapse after neoadjuvant chemotherapy [159] and disease-free survival in breast cancer, marking them as a prognostic marker [160]. CTCs offer insights into disease progression and treatment response in TNBC. Their association with residual cancer burden is an independent prognostic factor in patients with residual TNBC [161]. Case studies, like one utilizing a tumor-informed CTC test in an advanced TNBC patient, demonstrate CTCs’ utility in identifying therapeutic targets and monitoring treatment response [162]. Additionally, the role of CTCs in metastasis and chemotherapy effectiveness is under investigation, suggesting their therapeutic target potential [163].

**Figure 2:**
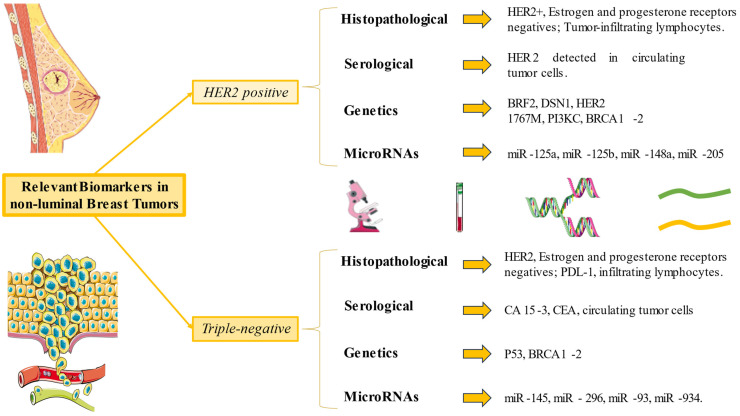
Summary of the most relevant biomarkers in metastatic HER2+ and triple-negative tumors.

In this sense, miRNAs and CTCs hold significant promise as prognostic and diagnostic tools in TNBC. Their association with survival, treatment response, and residual disease burden underscores their clinical importance in managing TNBC. The most relevant histological, serological, and genetic markers, as well as microRNAs of HER2+ and triple negative metastatic breast cancer, are concisely summarized in Figure 2.

## 7. Limitations

The integration of biomarkers in breast cancer treatment faces multifaceted challenges. Economically, the implementation of advanced detection technologies and targeted therapies based on biomarkers is costly, posing a significant barrier in resource-limited settings and developing countries. It should be noted that the interpretation of these biomarkers requires specialized training, a resource often lacking in less developed healthcare systems. Standardized methodologies are critical for the reliability of biomarker data, emphasizing the importance of uniform practices in sample collection and analysis to ensure data quality [164]. Furthermore, disparities in access to cutting-edge technologies for biomarker analysis lead to inequalities in breast cancer treatment across different regions or even in different parts of the same country [165]. Lastly, the use of genetic data in patient treatment raises ethical concerns, highlighting the need for comprehensive consent processes and robust data protection strategies to safeguard patient privacy and ensure the ethical handling of sensitive information [166].

In summary, while biomarkers offer a promising approach to breast cancer treatment, their integration is hindered by several technical and practical challenges. These include the high cost of advanced detection technologies, the need for specialized training in biomarker analysis, uneven access to necessary technology, and ethical concerns related to genetic data. Addressing these issues is crucial for leveraging biomarkers effectively in breast cancer care, requiring both technical advancements and policy considerations.

## 8. Conclusions

In conclusion, this article emphasizes the necessity of a multidisciplinary approach in managing metastatic breast cancer. It advocates for the integration of serological, histological, and genetic analyses, coupled with the examination of circulating tumor cells and microRNAs, thereby enhancing diagnostic accuracy. Moreover, this comprehensive strategy not only tailors treatments to individual patient profiles but also significantly improves the monitoring of disease progression. Consequently, these advancements herald a promising shift towards more precise and effective breast cancer care, signifying a pivotal moment in oncology.

## Figures and Tables

**Table 1:** This table summarizes the therapeutic implications of specific biomarkers in breast cancer, including clinical trial outcomes, side effects, challenges, and the corresponding references for each therapeutic agent.

Biomarker/Therapeutic Agent	Description	Clinical Trials and Outcomes	Side Effects/Challenges	References
**“DK4/6 Inhibitors”**				
Abemaciclib	Targets CDK4/6, effective in hormone receptor-positive breast cancer	MONARCH 2 [100], 3 [101], 1 [102], and E [103] trials showed improved progression-free survival	Diarrhea, thromboembolism, hematological toxicity, interstitial lung disease	[100,101,102,103]
Palbociclib	A potent CDK4/6 inhibitor	PALOMA-2 [104] and 3 [105] trials demonstrated effectiveness	Similar side effects as abemaciclib	[104,105]
Ribociclib	Another CDK4/6 inhibitor	MONALEESA-2 [106], 3 [107], and 7 [108] trials showed improvements	Comparable side effects to other CDK4/6 inhibitors	[106,107,108]
**“mTOR Inhibitors”**				
Everolimus	Inhibits mTOR signaling, effective in ER+ breast cancer	BOLERO-1 [110] and 2 [111] trials, mixed results in HER2+ cases	Neutropenia, stomatitis, diarrhea, alopecia	[110,111]
**“PI3K Inhibitors”**				
Alpelisib	Targets PI3K, effective in HR+/HER2- breast cancer with PIK3CA mutations	SOLAR-1 [113] trial showed improved progression-free survival	Hyperglycemia, maculopapular rash	[113]
**“Anti-HER2 Antibodies”**				
Trastuzumab	Targets HER2 receptor	Various trials (H0648g, M77001, BCIRG-006, HERA) [117]	Cardiotoxicity, pulmonary toxicity	[117]
Pertuzumab	Acts on a different domain of HER2	CLEOPATRA [118] and APHINITY [119] trials showed benefits	Similar side effects to trastuzumab	[118,119]
Margetuximab	Modified anti-HER2 antibody	SOPHIA trial [121] showed increased progression-free survival	Infusion-related reactions	[121]
**“PARP Inhibitors”**				
Olaparib	Targets PARP, effective in BRCA1/2 mutation breast cancer	OlympiAD [124] trial showed extended progression-free survival	Similar side effects to other PARP inhibitors	[124]
Talazoparib	Another PARP inhibitor	EMBRACA [125] trial, effective in gBRCAm HER2-negative advanced breast cancer	Comparable side effects to olaparib	[125]
